# Modeling Neuropsychiatric and Neurodegenerative Diseases With Induced Pluripotent Stem Cells

**DOI:** 10.3389/fped.2018.00082

**Published:** 2018-04-03

**Authors:** Elizabeth A. LaMarca, Samuel K. Powell, Schahram Akbarian, Kristen J. Brennand

**Affiliations:** ^1^Department of Neuroscience, Icahn School of Medicine at Mount Sinai, New York, NY, United States; ^2^Friedman Brain Institute, Icahn School of Medicine at Mount Sinai, New York, NY, United States; ^3^Department of Psychiatry, Icahn School of Medicine at Mount Sinai, New York, NY, United States; ^4^Graduate School of Biomedical Sciences, Icahn School of Medicine at Mount Sinai, New York, NY, United States; ^5^Medical Scientist Training Program, Icahn School of Medicine at Mount Sinai, New York, NY, United States; ^6^Department of Genetics and Genomics, Icahn School of Medicine at Mount Sinai, New York, NY, United States

**Keywords:** induced pluripotent stem cells, schizophrenia, Parkinson’s disease, neural induction, neural differentiation

## Abstract

Human-induced pluripotent stem cells (hiPSCs) have revolutionized our ability to model neuropsychiatric and neurodegenerative diseases, and recent progress in the field is paving the way for improved therapeutics. In this review, we discuss major advances in generating hiPSC-derived neural cells and cutting-edge techniques that are transforming hiPSC technology, such as three-dimensional “mini-brains” and clustered, regularly interspersed short palindromic repeats (CRISPR)-Cas systems. We examine specific examples of how hiPSC-derived neural cells are being used to uncover the pathophysiology of schizophrenia and Parkinson’s disease, and consider the future of this groundbreaking research.

## Introduction

Neuropsychiatric and neurodegenerative diseases are major contributors to the global burden of disease (1). Improved techniques to model these diseases and inform patient-specific treatment regimens are essential, as many current strategies have considerable limitations. Animal models cannot fully address the unique and complex pathophysiology of human neuropsychiatric disease, so findings are not always transferrable to the human condition. Human postmortem studies typically represent an advanced stage of disease and can be hindered by confounding factors, such as prescription and nonprescription drug use. These approaches also lack the capacity to predict patient-specific clinical outcomes to therapeutic drugs. Human-induced pluripotent stem cells (hiPSCs) are uniquely qualified to address these issues, as limitless patient-derived cells can be used to model human disease, screen drugs, and even generate tissues for transplantation. In this review, we will discuss how hiPSC technology has developed into a powerful platform for studying human neuropsychiatric diseases, focusing on schizophrenia and Parkinson’s disease (PD) as representative examples.

## A Brief History of hiPSC Research

In 2006, Shinya Yamanaka and his student Kazutoshi Takahashi demonstrated that iPSCs could be generated from mouse fibroblast cells *via* retroviral delivery of just four transcription factors—*c-Myc, Klf4, Oct3/4*, and *Sox2* (2). One year later, the first human iPSCs were generated independently by a number of groups (3, 4). The discovery of iPSCs was pivotal for the broad scientific community for several reasons: this was the first evidence that intact, adult cells could be reprogrammed to a pluripotent state; a new avenue of research was opened that was free of the moral and legal controversy surrounding embryonic stem cells (ESCs), and it was now feasible to generate large quantities of isogenic, patient-derived cells that can be differentiated into almost any cell type. Today, highly efficient, non-integrating reprogramming strategies (5–7) are widely used for studies of disease modeling and cell replacement therapy.

## Methods and Advances in Generating hiPSC-Derived Neural Progenitor Cells (NPCs) and Neurons

To understand how neural stem cells (NSCs), NPCs, and neurons can be generated from hiPSCs, it is helpful to first briefly review human neural development *in vivo*, outlined in Box 1.

**Figure 1:**
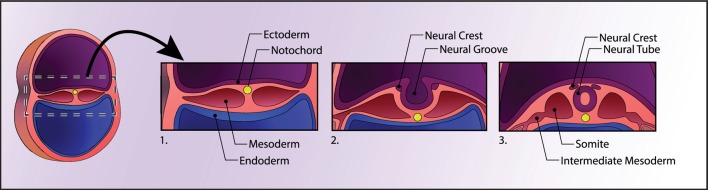
Human neural development. A cross section of the early embryo is depicted on the left. In the first panel, the three germ layers and the notochord are shown. The notochord signals the ectoderm above it to form the neural plate, which then folds into itself to form the neural tube, depicted in panel 3. See Box 1 for details.

Box 1 Approximately three weeks after fertilization, gastrulation occurs, in which the early embryo divides into the three germ layers; these will ultimately give rise to every organ in the body: the innermost layer, the endoderm, will form the epithelial lining of the gut and respiratory tract; the middle layer, the mesoderm, will form muscle and connective tissue; and the outermost layer, the ectoderm, will form the nervous system and the outer parts of the body, such as skin. Neurulation occurs next, which begins with the secretion of growth factors from a cylinder of mesodermal cells called the notochord. The notochord secretes Noggin, which inhibits a member of the transforming growth factor-β family, bone morphogenetic protein (BMP)-4, which would otherwise direct cells to form skin. Noggin thus allows cells to continue along their default neuroectodermal lineage and fold inward to form the neural tube (Figure 1). The inside of the neural tube goes on to become the ventricles of the brain, while the lining of the ventricles (i.e., the ventricular zone) gives rise to proliferative cells that migrate and differentiate in the intermediate layer. Various secreted morphogens along the dorsoventral and rostrocaudal axes of the neural tube allow cells to differentiate into functionally distinct and anatomically segregated classes of neurons, giving rise posteriorly to the spinal cord and anteriorly to the three major divisions of the brain: the forebrain (prosencephalon), which will later develop the cerebrum (telencephalon) and thalamic structures (diencephalon), midbrain (mesencephalon), and hindbrain (rhombencephalon), which will later develop the pons, cerebellum (metencephalon), and medulla (myelencephalon). Inductive signaling of sonic hedgehog and retinoic acid is involved in patterning the ventral axis of the neural tube, which give rise to motor neurons, while BMPs and Wnts are involved in patterning the dorsal axis of the neural tube, which primarily give rise to sensory neurons.

### Directed Differentiation

Aggregates of human pluripotent stem cells known as embryoid bodies will spontaneously differentiate into cells closely resembling those of all three embryonic germ lineages (8–10) and will further differentiate into neural-like cells at a low efficiency. Neural differentiation can be promoted by either inclusion of growth factors that promote neural differentiation or withdrawal of those factors that prevent it (i.e., mimicking *in vivo* cell differentiation) in a process known as “directed differentiation.” As mentioned in Box 1, the transforming growth factor-β protein, bone morphogenetic protein (BMP), directs cells away from a neuroectodermal lineage; this occurs *via* intracellular signaling to the nucleus by SMAD proteins (11). In 2004, it was demonstrated that treatment with the BMP antagonist Noggin (12) dramatically improved neural differentiation. Noggin-mediated SMAD inhibition was later improved by the addition of a second SMAD inhibitor, SB431542, resulting in a technique known as dual SMAD inhibition to promote neural differentiation (13). This technique is highly efficient, leading to 80% conversion to NPCs (13).

By default, NPCs generated by dual SMAD inhibition are characteristic of a rostral, or more primitive, identity (14). Caudalization can be achieved by treatment with an inhibitor of glycogen synthase kinase 3 (GSK3) to activate Wnt signaling; the addition of sonic hedgehog and/or fibroblast growth factor 8 can further direct cells toward a ventral mesencephalic lineage, yielding midbrain dopaminergic (DA) neurons (15) at yields of up to 80% [well reviewed in Ref. (16)]. These DA neurons express transcriptional profiles best aligned with midbrain DA identity, display appropriate electrophysiological activity, and restore lost functionality in animal models of PD [e.g., Ref. (15, 17–20)].

Directed differentiation protocols have several potential limitations, including a protracted differentiation period, the need for costly growth factors that can vary from lot to lot, and the generation of a heterogeneous population of cells that most closely resemble fetal forebrain neurons (21, 22). These issues can be partially circumvented *via* direct conversion of somatic cells into neurons in a strategy known as “neuronal induction.”

### Neural Induction

Neuronal induction was first achieved by overexpressing a set of three transcription factors known as the BAM factors, *Brn2* (also known as *Pou3f2*), *Ascl1*, and *Myt11*, in mouse fibroblasts (23, 24). The same group later showed that this strategy is also effective for human fibroblasts when combined with the transcription factor *neurogenic differentiation factor 1* (*NEUROD1*) (25), although these induced neurons were unable to form synapses without a mouse cortical neuron substrate unless expressed with specific microRNAs (26, 27). Specific neuronal subtypes (28–31) or NPCs (32–35) can be induced through the addition of cell-type-specific transcription factors. More recently, human astrocytes have been induced *in vitro*, and mouse astrocytes *in vivo*, into mDAergic neurons through overexpression of *NeuroD1, Ascl1, Lmx1a*, and the microRNA miR218 (36).

In 2013, Zhang et al. showed that it was possible to induce nearly 100% pure populations of neurons from ESCs or hiPSCs *via* forced overexpression of just a single transcription factor, either *Neurogenin2* (*NGN2*) or *NEUROD1* (37), yielding functional synapses within just two weeks. Subsequently, Ho et al. applied this technique to induced neurons from hiPSC-derived NPCs, which are easier to culture and more amenable to high-throughput drug screens (38).

### Three-Dimensional (3D) “Mini-Brains” in a Dish

Two-dimensional neural culture systems have vastly expanded our understanding of neurological development and disease, but lack the ability to accurately model the cytoarchitecture and network connectivity of the 3D human brain. Within the last decade, 3D neural culture systems have begun to address this issue and are leading to improved disease modeling approaches.

**Figure 2:**
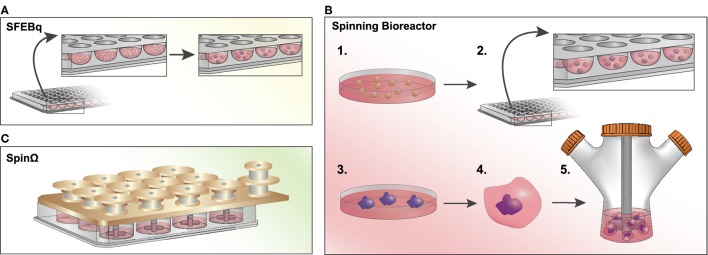
Methods to generate brain organoids *in vitro*. **(A)** Serum-free, floating culture of embryoid bodies with quick reaggregation (SFEBq): pluripotent stem cells (PSCs) are dissociated and resuspended in a low-adhesion, U-bottomed 96-well plate in serum-free culture. Cells will quickly reaggregate (within an hour) into uniformly sized masses and will differentiate into neural progenitor cells within ~10–12 days after plating in N2-supplemented media. **(B)** Spinning Bioreactor: 1. PSCs are dissociated and 2. cultured in a low-adhesion plate in hES media with low bFGF and a ROCK inhibitor. 3. Embryoid bodies are cultured in neural induction medium and begin to form neuroepithelial tissues. 4. Tissues are transferred into Matrigel droplets and four days later are 5. transferred to a spinning bioreactor with differentiation media. **(C)** SpinΩ: A spinning device is fitted over a standard 12-well culture plate, with 13 interconnecting gears driven by a motor agitating the culture media. Similar to the spinning bioreactor approach, cells are embedded in Matrigel droplets prior to spin culture. This technique significantly reduces the cost and space constraints associated with spinning bioreactors.

The first neural organoid-like structures derived from PSCs were developed using a serum-free, floating culture of embryoid bodies (SFEB) (39), which suffered from low efficiency and inconsistent aggregate sizes. Seeding dissociated mouse ESCs into U-bottomed, low-adhesion plates led to quick reaggregation (SFEBq) of uniformly sized masses and consistent differentiation into cortical neuron-like cells (Figure 2A) (40). By seeding cells in a V-bottom plate in the presence of a rho kinase (ROCK) inhibitor to reduce apoptosis, the SFEBq technique is also suitable for use with human PSCs (41). Importantly, the authors showed that 3D structures were capable of self-organizing into apicobasally polarized neuroepithelial structures. With the addition of rostral-neutralizing factors, organoids can produce polarized radial glia, intermediate progenitors, and both early- and late-born cortical neurons that display evidence of synapse formation (including vesicular glutamate transporter vGLUT1-positive excitatory neurons and dorsal telencephalic-like GABAergic interneurons) (21). Mimicking the laminin-rich basement membrane of epithelial structures with Matrigel improves organoid self-organization (42).

In a seminal study by Lancaster et al., large and complex cerebral organoids were generated by seeding neuroectodermal aggregates embedded in Matrigel droplets in a spinning bioreactor to enhance nutrient absorption (Figure 2B) (43). After less than a month, the organoids developed a regional specification analogous to the early fetal brain *in vivo*. In an attempt to reduce the large volumes and space constraints associated with the spinning bioreactor approach, Qian et al. developed SpinΩ, a mini-bioreactor to generate organoids from hiPSCs (Figure 2C) (44). This study yielded organoids with a human-specific radial glial cell outer layer and all six layers of the cortex clearly defined, as well as several major interneuron subtypes expressing parvalbumin, nNOS, or somatostatin. Furthermore, the authors were able to generate more homogeneous forebrain structures with the addition of a pre-patterning step to initially instruct cell fate direction (44). Cortical spheroids can also be generated without spinning bioreactors or Matrigel through dual SMAD inhibition followed by culture with a neuron differentiation-promoting medium (45). Such spheroids are more homogeneous, containing excitatory pyramidal cell-like deep and superficial cortical neurons, as well as fully integrated astrocytes.

To model the impact of vasculature, hESC-derived NPCs were seeded on peptide-functionalized polyethylene glycol hydrogels, followed by addition of hESC-derived microglial and vascular cells in a time frame that corresponded to the integration of these cells during fetal brain development (46). These organoids formed a vascular network and incorporated both ramified and amoeboid microglial cells, leading to a highly similar transcriptional profile to that of the early fetal brain. Recently, Lancaster et al. combined traditional organoid generation and bioengineering techniques to physically guide 3D differentiation with microfilaments, leading to an increased fidelity of forebrain-like cells and capacity for radial glial migration (47).

Organoids have remarkably similar gene expression patterns to their *in vivo* fetal brain counterparts (48, 49) and are capable of spatial patterning and cell fate commitment in a similar time frame to that seen *in vivo*. It is possible to optically clear 3D neural cultures in order to visualize their internal network connectivity while reducing the time and labor associated with standard tissue sectioning and histology, leading to greater scalability (50). Several limitations do exist, however, primarily concerned with the lack of true patterning axes *in vitro*, precluding the development of more complex self-organizing structures. In addition, although successive techniques have reduced inter-sample variability, this still remains a major limiting factor for assays that require homogeneity among samples such as drug screens. There is a need for future studies to address these issues, potentially by integrating novel bioengineering strategies. For a more detailed review of 3D neural culture techniques and considerations, readers are referred to an excellent review by Kelava and Lancaster (51). Overall, brain organoids seem to faithfully represent human corticogenesis and as such are excellent platforms upon which to study complex neurodevelopmental diseases.

### Clustered, Regularly Interspersed Short Palindromic Repeats (CRISPR)

**Figure 3:**
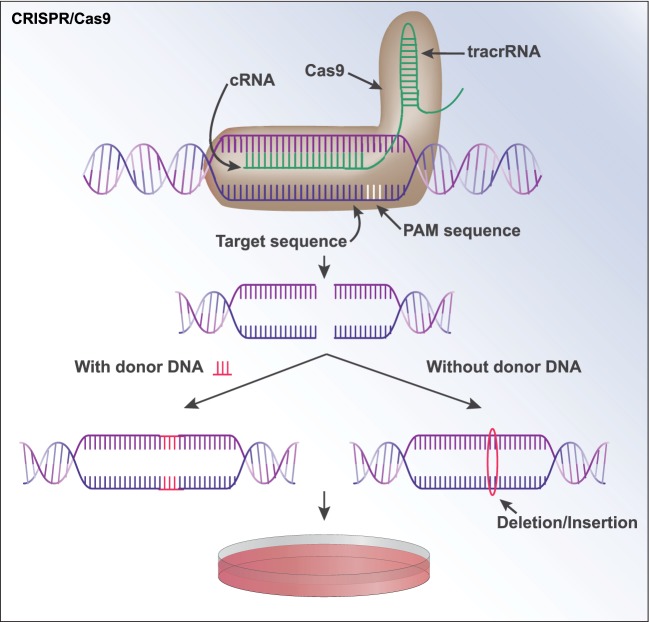
CRISPR/Cas9. A sequence of DNA can be modified by CRISPR/Cas9 through the formation of a guide RNA (gRNA) that contains an RNA complementary to the target DNA sequence. The gRNA additionally contains a noncoding transactivating RNA (tracrRNA) that functions as a “scaffold” to bind to Cas9. The gRNA and Cas9 form a complex and is brought to the target sequence, where Cas9 binds to a specific protospacer adjacent motif (58). Cas9 then cuts both strands of the DNA. If the user supplies a “donor sequence,” it will serve as a repair template and be incorporated at the break site in a process known as homology-directed repair. If no donor sequence is provided, indels are often created as the cell repairs the double-strand break *via* non-homologous end joining. This can knock out the gene of interest, as the indels are often capable of creating frameshift mutations. The modified sequence can now be expanded in cells in culture.

The (CRISPR)/Cas (CRISPR-associated protein) system enables targeted manipulation of genetic material. Initially identified as a key immune defense mechanism in some bacterial species and Archaea (52–54), the CRISPR/Cas9 system involves the production of CRISPR RNAs that bind to Cas endonucleases and cleave invading genetic material (55). Of particular interest to current applications is the type II CRISPR/Cas9 system in which a noncoding transactivating (tracrRNA) facilitates binding of complexes consisting of CRISPR RNAs and Cas9 to target loci (56, 57) (Figure 3). In the presence of a protospacer adjacent motif (58) that is compatible with Cas endonuclease binding, this complex cleaves the genetic material to which it is bound (59, 60). In modern applications, the tracrRNA and cRNA are typically synthesized as a single “guide RNA” (gRNA) (55). A commonly used endonuclease is the *Streptococcus pyogenes* Cas9 (Sp Cas9) (61) but several others exist [reviewed in Ref. (62)]. In addition to those enabling targeted cutting of genetic material, novel Cas9 endonucleases have been generated for further applications. “Nickase Cas9” (nCas9) is an endonuclease in which one of the two nuclease domains has been mutated such that only one of the target DNA strands is cut (63), which enhances target specificity by nature of the fact that only adjacent nCas9-mediated cuts generate the desired double-stranded break (63–65). Furthermore, the mutation of both nuclease domains creates a null or “dead” Cas9 (66) to which several transcriptional effectors can be fused for the modulation of gene expression at loci of interest (67–69).

The CRISPR/Cas9 system can be used in a variety of ways to study neurological and psychiatric diseases in stem cell-based models (70, 71). One particularly promising application is the manipulation of genetic material to explore the role of putative allelic variations in neurological and psychiatric conditions. Introduction of a PD-associated single nucleotide polymorphism (SNP) in an enhancer region of alpha-synuclein (SNCA) altered histone posttranslational modifications at this site and increased the expression of SNCA (72). In the case of Fragile X Syndrome, deletion of the disease-causing trineucleotide repeat expansion of CGG in the FMR1 gene in hiPSC neurons derived from patients with FXS resulted in normalized expression of FMR1 (73). The generation of NPCs and neurons (74) and cerebral organoids (75) with heterozygous deletions in chromodomain helicase DNA-binding protein 8 (CHD8), a gene implicated in ASD-associated *de novo* mutations (76–78), provided support for a role of Wnt signaling in ASD pathophysiology. Beyond genomic-editing approaches, a nuclease null or dead Cas9 fused to transcriptional regulators can be employed to alter the expression of several genes implicated in neuropsychiatric disease (79, 80). Overall, these reports are robust demonstrations of the feasibility of CRISPR/Cas9 approaches in disease modeling with hiPSC-derived neurons.

## Advances in the Field of Neuropsychiatric hiPSC-Based Disease Modeling

### Schizophrenia

Schizophrenia is a severe, complex genetic psychiatric disorder that affects approximately 1% of the population worldwide, primarily characterized by positive, negative, and cognitive symptom domains (81). Large-scale genetic studies have identified 108 common variant loci (82) and eight copy number variations (83) that are significantly associated with schizophrenia. Here, we will discuss how hiPSCs have been used to dissect the pathophysiology associated with genetic risk for schizophrenia, focusing on well-documented phenotypic consequences.

#### 22q11.2

The 22q11.2 region of the genome is flanked by two low-copy repeat segments that are susceptible to non-allelic homologous recombination. Deletions in this region are often at these “breakpoints” and typically occur *de novo*, as a result of aberrant recombination during meiosis (84). 22q11.2 deletion as a risk allele for schizophrenia has been confirmed at genome-wide significance in a cohort of ~40,000 cases and controls, and interestingly, duplications of 22q11.2 were suspected to have a protective effect in controls (83, 85). Deletions of 22q11.2 have also been implicated in autism spectrum disorders, attention-deficit/hyperactivity disorder, and anxiety and affective disorders (86).

Abnormal gene (87) and microRNA expression (88) occurs in hiPSC-derived 22q11.2 del neurons, with an enrichment for genes and microRNA targets involved in neuropsychiatric disorders. hiPSC-derived 22q11.2 neurons further show impaired ability to differentiate into neurons, abnormal neurite outgrowth, migration, neuron-to-glia ratios, and have smaller neurosphere sizes (89). The downstream targets of DGCR8 (contained in the 22q11.2 region and essential for microRNA biosynthesis), miR-106a, and the miR-17/92 cluster were reduced in patient-derived neurospheres; this finding implicated their target p38a, which drives glial differentiation, and could potentially explain the abnormal neuron-to-glia ratios observed (89). In addition to dysregulated gene expression, 22q11.2 del hiPSC neurons have higher levels of the LINE-1 retrotransposon in synaptic genes, consistent with observations in 22q11.2 del patient postmortem brains (90). To date, the lack of genetically engineered rescue of 22q11.2 deletions in hiPSC neurons has limited efforts to identify the causal gene(s) within the region responsible for these molecular and cellular phenotypes.

#### Disrupted in Schizophrenia 1 (DISC1)

The discovery of the *DISC1* gene dates back to 2000, following a nearly 30-year longitudinal study of a Scottish family with a history of psychiatric illness (91, 92). Researchers identified a balanced translocation between chromosome 1 and chromosome 11 in 37 of 69 biologically related, karyotyped family members, 29 of whom were diagnosed with a psychiatric illness (93). This translocation was found to disrupt two genes on chromosome 1, *DISC1* and *DISC2* (91). DISC1 is an important regulator of embryonic and adult neurogenesis, neurite outgrowth, and synapse formation (94–96), serving as a cytoskeletal scaffolding protein and a modulator of GSK3β and Wnt signaling (97–99).

Human-induced pluripotent stem cell-derived neurons from two related patients with a frameshift mutation in *DISC1* had impaired synaptic vesicle release relative to two clinically unaffected family members without the mutation (100). Using transcription activator-like effector nucleases (TALENs), the authors were able to reverse the respective synaptic phenotypes by editing in the mutation into control lines and correcting it in patient lines, thereby establishing a direct link between *DISC1* mutation and synaptic dysfunction. DISC1 regulation at the synapse has been further elucidated by Kassan et al., who found that both mouse and hiPSC-derived neurons have an increased expression of DISC1 and other synaptic proteins when caveolin-1 (Cav-1) is overexpressed under the control of a neuron-specific synapsin promoter and decreased when Cav-1 is knocked out (KO) (101). The authors restored the expression of DISC1 and the other synaptic proteins by re-introducing the synapsin-driven Cav-1 vector into Cav-1-KO neurons.

In addition to synapse function, DISC1 disruption is also deleterious for normal processes involved in mitosis and neural cell fate commitment. DISC1 regulates the attachment of Ndel1 to the kinetochore during mitosis, and the t(1; 11) translocation disrupts this binding complex (102). By introducing a peptide that specifically interferes with the binding of Ndel1 to DISC1 (but does not affect the binding of either DISC1 or Ndel1 to other proteins) in hiPSC-derived forebrain organoids, Ye et al. showed that this complex is important for the proliferation of NSCs, and its disruption leads to delayed cell-cycle progression (103). In line with this finding, hiPSC-derived forebrain organoids from a schizophrenia patient containing a frameshift mutation in the C-terminal region of DISC1 showed prolonged cell-cycle progression during mitosis. The role of DISC1 in neural cell fate commitment was demonstrated by Srikanth et al., who used TALEN- and CRISPR–Cas9 to mutate *DISC1* in hiPSCs (104), reporting aberrant neural differentiation and increased Wnt signaling. Given its many protein-binding interactions, understanding the mechanism by which this very rare mutation impacts neuronal function may ultimately inform a larger understanding of schizophrenia risk.

#### Antipsychotic Drug Modeling

Human-induced pluripotent stem cell-derived neural cells can be used with relative ease to probe the efficacy and mode of action of pharmaceuticals both for large populations and for individual patients. In the first study demonstrating the feasibility of using hiPSC-derived neural cells to model schizophrenia and to study antipsychotic drug effects *in vitro*, deficits in neuronal connectivity were partially reversed by culture with the first-generation antipsychotic loxapine, which additionally raised levels of several glutamate receptors in the patient cell lines (105). Similarly, Asada et al. found that antipsychotic drugs promote neural differentiation in hiPSC-derived cells (106). In two complementary studies, Paulsen et al. showed that hiPSC-derived neural cells had significantly increased levels of extramitochondrial oxygen consumption and levels of harmful reactive oxygen species (ROS) compared to controls (107), together with elevated levels of potassium and the putative antioxidant zinc (108). Treatment with the mood stabilizer Valproate increased extramitochondrial oxygen flow and normalized both ROS and zinc levels in the patient NPCs.

It may also prove possible to probe the underlying mechanism of discordant antipsychotic drug response with hiPSC-derived neural cells. hiPSC-derived neurons generated from a pair of monozygotic twins with treatment-resistant schizophrenia (only one of whom responded to clozapine) showed unique gene expression patterns, particularly with respect to homophilic cell adhesion, in the responder relative to the non-responder both basally and during treatment with clozapine (109). Consistent with this, Maschietto et al. found many differentially expressed genes in hiPSC-derived NPCs from a patient with treatment-resistant schizophrenia, many of which are also involved in cell adhesion (110).

Overall, these studies (outlined in Table 1) demonstrate the clinical utility of hiPSC-derived neural cells in the study of both schizophrenia and its treatment. More personalized preclinical models could mitigate costly clinical trial failures, as individual patient-derived hiPSC neurons could be treated with arrays of different antipsychotics to test drug efficacy before prescribing to patients.

**Table 1:** Studies of human-induced pluripotent stem cell models of schizophrenia.

Mutation	Subjects	Reprogramming method	Cell type	Phenotype	Reference
22q11.2 del	2 cases, 2 controls	Retroviral	Neurons	High L1 copy number.	(90)

22q11.2 del	6 cases, 6 controls	Plasmids	Neurons	Differential expression of miRNAs putatively involved in neurological and neuropsychiatric disorders.	(88)

22q11.2 del	8 cases, 7 controls	Plasmids	Glutamatergic/GABAergic neurons	Reduced expression of most genes in the 22q11.2 region. Altered expression of genes in pathways involved in apoptosis, cell cycle/survival, and MAPK signaling.	(87)

22q11.2 del	2 cases, 3 controls	Retroviral	Neurons and neurospheres	Neurosphere size, neural differentiation efficiency, neurite outgrowth, cellular migration, and neuron-to-glia ratio reduced. Reduced miRNA expression. Upregulation of p38α.	(89)

Disrupted in schizophrenia 1 (DISC1) (introduced *via* clustered, regularly interspersed short palindromic repeat (CRISPR)/TALEN)	6 wild-type (WT) clones, 6 WT/mut clones, 13 mut/mut clones	Lentiviral	Neurons, NPCs	Decreased DISC1 protein levels due to nonsense-mediated decay of long splice variants. Increased Wnt signaling altered expression of fate markers.	(104)

DISC1 frameshift	2 cases (SCZ/MDD), 2 controls. 1 unrelated control	Episomal vectors	Forebrain neurons	Reduction of synaptic boutons and deficits in synaptic vesicle release.	(100)

DISC1 translocation	1 case, 2 unrelated controls	Episomal vectors	Forebrain organoids	Disrupted DISC1/Ndel1 interaction; cell-cycle deficits/delayed mitosis.	(103)

Neuronal cav-1 overexpression	SynCav1-transfected neurons	Retroviral	Neurons	Increased expression of DISC1 and synaptic plasticity proteins.	(101)

Unknown	Monozygotic twins, differential response to clozapine	Plasmids	Neurons	Differential expression of genes encoding homophilic cell adhesion molecules.	(109)

Unknown	4 cases, 7 controls	Lentiviral	Neurons	Decreased neuronal connectivity, neurite number, PSD-95, and glutamate receptor expression. Altered expression of cAMP and Wnt-signaling pathway components. Ameliorated after loxapine treatment.	(105)

Unknown	1 case, 1 control	Retroviral	NPCs	Increase in extramitochondrial oxygen consumption, elevated levels of reactive oxygen species, ameliorated with valproic acid.	(107)

Unknown	1 case, 2 controls	Retroviral	NPCs	Elevated potassium and zinc levels, ameliorated with Valproate.	(108)

Unknown	4 cases, 6 controls	Lentiviral	NPCs and neurons	Abnormal levels of genes and proteins related to cytoskeletal remodeling and oxidative stress. Altered migration and increased oxidative stress.	(22)

*het, heterozygous; mut, mutant; OPC, oligodendrocyte progenitor cell; NPC, neural progenitor cell; NSC, neural stem cell; del, deletion, SCZ, schizophrenia; MDD, major depressive disorder*.

### Parkinson’s Disease

Parkinson’s disease is a neurological disorder characterized by hallmark motor symptoms of rigidity, tremor, bradykinesia, and postural instability (111). In addition to these, numerous nonmotor symptoms can be observed, including cognitive impairment, mood disorders, psychosis, fatigue, autonomic dysfunction, olfactory abnormalities, and gastrointestinal dysfunction (112). During the many decades of active research on this disorder, a persistent challenge has been the lack of model systems that allow the examination of cellular and molecular deficits in living neurons from patients with PD and an inability to perform meaningful drug screens on human tissue. In this section, we review many studies that have addressed these problems by generating hiPSC-derived neurons from patients with defined genetic mutations associated with PD. These findings have demonstrated a variety of pathological disturbances in PD and have paved the way toward therapeutic innovation.

#### Leucine-Rich Repeat Kinase 2 (*LRRK2*)

Mutations in *LRRK2* are associated with both idiopathic and familial PD (113, 114). In the first report describing the generation of hiPSCs from a patient with a *LRRK2* G2019S mutation, differentiated DA neurons displayed an abnormal accumulation of alpha-synuclein, altered expression patterns of genes involved in oxidative stress, and an increased susceptibility to chemical stressors (115). Neurons generated from patients with the same *LRRK2* mutation or an R1441C mutation exhibited dysfunction of several mitochondrial pathways, some of which could be rescued with compounds, including an LRRK2 inhibitor, identified from pharmacologic screens (116). A similar report showed mitochondrial DNA damage in DA neurons with either *LRRK2* mutation, which could be alleviated with gene correction (117). Furthermore, the targeted inhibition of mitochondrial fission prevented elevated autophagy and mitochondrial pathology in *LRRK2* neurons (118).

Deficits in *LRRK2*-mediated arrest of dysfunctional mitochondria in G2019S-mutant neurons may be mediated by the outer mitochondrial membrane protein Miro, as G2019S-mutant neurons exhibited abnormal retention of Miro and persistence of dysfunctional mitochondria, leading to neurodegenerative phenotypes that could be improved through reduction in Miro levels (119). A later report also found abnormal mitochondrial dynamics that were associated with a decreased Sirtuin2 activity (120). In others, midbrain DA neurons derived from patients with idiopathic and *LRRK2* G2019S PD demonstrated disrupted autophagy and neurite growth patterns (121, 122). NPCs generated from *LRRK2* G2019S patients degenerated more compared to wild-type controls in a passage-dependent manner, which was associated with progressive nuclear damage and the acquisition of epigenetic markers associated with aging (31). In addition, neurons generated from patients with *LRRK2* mutation and sporadic PD had DA neuron-specific hypermethylation and alteration in gene expression profiles, including aberrant expression of transcription factors with potential relevance to PD (123).

Genetic correction experiments ameliorated the neurodegenerative phenotypes in *LRRK2* neurons and provided evidence for the involvement of the ERK-signaling pathway in *LRRK2* mutation-mediated neuropathology; furthermore, disease-related neuronal dysfunction could be improved by the inhibition of the ERK pathway (124). In an alternative approach, the generation of sensory neurons from *LRRK2* G2019S hiPSCs implicated abnormal neurite outgrowth and altered calcium responses to depolarization as pathologies driving some of the nonmotor symptoms of PD (125), and these abnormalities could be alleviated with pharmacologic inhibition of LRRK2. Finally, both R1441G- and G2019S *LRRK2*-mutated neurons exhibited altered NF-kB transcriptional responses (126), and gene expression profiles have also been explored in *LRRK2* G2019S neural organoids (127). Beyond the *LRRK2* mutations already mentioned, hiPSC lines have also been generated from patients with different *LRRK2* mutations, including R1398H, R1628P, S1647T, and N551K (128–131). Overall, these studies provide robust demonstration of the ability of hiPSC approaches to reveal disease-associated pathologies in a mutation-specific manner.

#### PINK1 and Parkin

Mutations in both PTEN-induced putative kinase 1 (*PINK1*) (132) and *Parkin* (133) are implicated in autosomal recessive forms of familial PD. These two proteins interact in a mitochondrial-autophagy pathway in which PINK1 recruits Parkin to damaged mitochondria (134, 135) and Parkin subsequently ubiquitinates mitochondrial proteins, targeting them for autophagy (136, 137).

Generation of hiPSC-derived midbrain DA neurons from patients with Parkin mutations revealed an increased oxidative stress and a spontaneous dopamine release mediated by increased transcription of monoamine oxidases (138). Later reports found accumulation of alpha-synuclein and abnormalities in mitochondrial dynamics in *PINK1-* and *PARK2*-mutant hiPSC-derived neurons (139, 140). *PINK1*-mutant neurons exhibited a deficient recruitment of Parkin (141) and an altered Parkin-mediated ubiquitination in response to mitochondrial depolarization (142). Mitochondrial abnormalities were also seen in DA neurons from *PARK2* patient-specific mutant and isogenic PARK2-knockout hiPSC lines (143). Different investigators have also shown a heightened sensitivity to metal toxins including manganese (144) and copper (145) in PARK2-mutant hiPSC-derived NPCs. Both TH^+^ and non-TH^+^ neurons with PARK2 mutation have several alterations in neurite morphology (146), and a later report documented disrupted microtubule stability (147). *Parkin*-mutant neurons also exhibit abnormalities in endosomal processes and trafficking (148), as well as disrupted calcium shuttling between mitochondrial and endoplasmic reticulum (ER) (149).

#### Alpha-Synuclein

Alpha-synuclein, encoded by the *SNCA* gene is the key component of Lewy body inclusions, which are a pathological hallmark of PD (150); numerous mutations in *SNCA* have been associated with the disease (151–153). Human ESC lines with point mutations in *SNCA*, isogenic controls, and SNCA-mutation-associated PD patient hiPSC lines have been generated to provide a platform to study perturbations in alpha-synuclein in a targeted fashion (154). Cortical neurons generated from hiPSC lines of patients with alpha-synuclein mutations exhibited increased nitrosative and ER stress and accumulation of ER degradation substrates; following this observation, a small molecule identified in a yeast screen reversed some of these abnormal phenotypes (155). On the other hand, DA neurons generated from alpha-synuclein-mutant hiPSC patient cells also showed nitrosative stress, which led to Mef2c nitrosylation and a subsequent decreased activity of the neuroprotective peroxisome proliferator-activated receptor gamma coactivator-1alpha (PGC1alpha) pathway; such dysregulation led to mitochondrial damage and subsequent apoptosis (156). Neurons generated from hiPSC lines with a variety of SNCA mutations contained altered ratios of alpha-synuclein tetramers to monomers, providing further evidence for the abnormal accumulation of alpha-synuclein as a pathologic hallmark of PD (157). Strikingly, the introduction of a PD-associated SNP in an enhancer region of SNCA enhanced H3K27ac at this region and led to an increased alpha-synuclein expression and altered transcription factor binding in hiPSC-derived neurons (72).

Triplication of *SNCA* results in a completely penetrant and pernicious form of PD with dementia (158), and midbrain DA neurons generated from an SNCA triplication patient line produced approximately double the normal levels of alpha-synuclein (159). These types of cells also exhibit an increased susceptibility to oxidative stress (160) and lysosomal dysfunction (161). Similar SCNA triplication cell lines also had diminished capabilities to differentiate into both GABAergic and DA neurons, with a corresponding decrease in differentiation-promoting genes (162). Furthermore, the same study documented abnormal cation currents, decreased connectivity, and dramatically altered action potential profiles in neurons with SNCA triplication (162).

Overall, these studies of patient-specific PD hiPSC neurons (outlined in Table 2) have revealed an array of similarities and differences between DA neurons carrying different PD genes, suggesting that a targeted genotype-first therapeutic approach might also advance treatments for PD.

**Table 2:** Studies of human-induced pluripotent stem cell (hiPSC) models of Parkinson’s disease (PD).

Mutation	Subjects	Reprogramming method	Cell type	Phenotype	Reference
SNCA A53T	1 case (later corrected to an isogenic control)	Lentiviral	Forebrain NPCs and neurons	Altered levels of alpha-synuclein tetramers and cell toxicity	(157)

SNCA triplication	1 case, 1 unaffected first-degree relative	pMX vectors	mDA neurons	Increased alpha-synuclein levels	(159)

SNCA triplication	Lines from patient and unaffected relative	Retroviral	Multiple neuronal types	Impaired maturation and differentiation	(162)

LRRK2 G2019S and R1441C	5 R1441C heterozygous, 1 homozygous G2019S, 6 G2019S	Retroviral	Neural cells	Mitochondrial dysfunction that can be reversed with gene correction	(117)

LRRK2 G2019S	2 homozygous G2019S, 1 homozygous G2019S	Lentiviral	mDA neurons	Improper dynamics of damaged mitochondria	(119)

LRRK2 G2019S	2 homozygous, 1 heterozygous, 3 unaffected controls	Lentiviral	mDA neurons	Potential role for sirtuin dysfunction in disease-associated mitochondrial damage	(120)

LRRK2 G2019S	2 homozygous, 1 heterozygous, 3 unaffected controls	Lentiviral	Sensory neurons	Neurite aggregation and calcium dysfunction	(125)

LRRK2 G2019S and Sporadic PD	4 heterozygous LRRK2, 6 sporadic cases, 4 controls	Retroviral	mDA neurons	Altered patterns of genome methylation and gene expression	(123)

LRRK2 G2019S	4 cases, 3 controls	Episomal vectors	Neuroectodermal spheres	Altered patterns of gene expression	(127)

PARK2	2 del lines, 2 unaffected controls	Lentiviral	mDA neurons	Increased oxidative stress and altered dopamine metabolism	(138)

PARK2	1 del line; 1 control line	Retroviral	Mix neural cells	Increased oxidative stress, mitochondrial dysfunction, and alpha-synuclein accumulation	(139)

PARK2 and PINK1	PARK2 V324A homozygous; PINK1 Q456X; 2 controls	Retroviral	mDA neurons	Alpha-synuclein accumulation and mitochondrial dysfunction	(140)

PINK1	1 homozygous PINK1 V170G	Retroviral	DA neurons	Mitochondrial dysfunction	(142)

PARK2	Various mutations	Sendai viral vectors	mDA neurons	Mitochondrial dysfunction	(143)

*DA, dopamine; mDA, midbrain dopamine; NPC, neural progenitor cell; NSC, neural stem cell; del, deletion*.

## Challenges of hiPSC Disease Modeling and Future Perspectives

Despite recent advances in hiPSC differentiation protocols, several obstacles still remain. hiPSC-derived neurons are currently unable to mature past a fetal stage, which can be limiting for studies of diseases that primarily affect the adult brain, such as PD. In order to address this issue, a consensus must first be reached concerning the measurable hallmarks that define cellular aging. Lopez-Otin et al. proposed nine such hallmarks that can be used as screens for cellular aging, including altered intracellular communication, cellular senescence, deregulated nutrient-sensing, epigenetic alterations, genome instability, loss of proteostasis, mitochondrial dysfunction, stem cell exhaustion, and telomere attrition (163).

The overexpression of progerin, a protein involved in the premature aging disorder Hutchinson–Gilford progeria, in hiPSC-derived mDA neurons leads to several age-related deficits, such as mitochondrial abnormality and AKT deregulation (164). Although progerin overexpression is unable to recapitulate all aspects of normal aging, it may prove especially useful for modeling late-onset disorders *in vitro*. Neural induction strategies can be used to accelerate differentiation, and coculture of neurons with astrocytes (165) or astrocyte-conditioned medium (166, 167) has been shown to promote electrophysiological maturation. More studies are needed to assess whether these techniques are capable of accelerating other characteristics of cell maturation, such as late-stage neuronal markers and morphology (168).

Sample heterogeneity between differentiation strategies (169, 169) and laboratories (170) remains a large issue for hiPSC research. Optimizing and standardizing differentiation protocols across laboratories are thus necessary to permit the generation of accurate, reproducible, and scalable data. Several large-scale hiPSC initiatives, such as the Innovative Medicines Initiative EU Centralized iPSC Repository, the New York Stem Cell Foundation, the California Institute for Regenerative Medicine, Kyoto University Center for iPS Cell Research & Application, Progenicyte, HipSci, StemBANCC, and the National Institute of Mental Health (171) have been founded to create biorepositories of well-characterized hiPSC lines from control and patient groups for widespread distribution to researchers and clinicians around the world. Such large-scale initiatives will help to increase the statistical power of hiPSC-based studies, which have historically suffered from low sample sizes and variability among samples and replicates. Automated culture systems can also help to reduce sample variability (172). Furthermore, two recent studies demonstrated the need for increased donor lines in hiPSC-based studies in order to minimize sample variability (168, 169), even at the expense of reducing the number of replicated hiPSC lines per individual.

Since the inception of hiPSC technology, researchers have made tremendous progress in engineering these *in vitro* systems to more closely resemble the molecular and electrophysiological properties of human cells *in vivo*. Recently, several studies have reported improved culture techniques that permit enhanced network-wide electrophysiological properties in hiPSC-derived neural cells (173, 174). In addition, Kuijlaars et al. showed that coculture with human primary astrocytes leads to increased neuronal network activities, such as bursting frequency and calcium oscillation synchronization over time (175). Other advances in the area of biomaterials (176, 177) are improving the efficiency, scalability, and fidelity of hiPSC-derived neuronal differentiation strategies (177). As these innovations continue to improve hiPSC technology, researchers will be better equipped to model the complex phenotypes associated with neuropsychiatric and neurodegenerative illnesses.

The CRISPR/Cas system is undoubtedly revolutionizing hiPSC research. Off-target effects present a major drawback, but can be mitigated by optimizing gRNA design or by using high-fidelity alternatives to the classic Cas9 endonuclease (178). More importantly, the commonly used lentiviral delivery strategy is highly efficient but prone to random genomic integration, limiting its clinical applicability. Alternative, non-integrating approaches have been proposed, such as adeno-associated viruses and nonviral methods (178–180), which may provide safer alternatives that can be used in a clinical setting. CRISPR-based clinical trials aimed at treating human immunodeficiency virus, Epstein–Barr-associated malignancies, gastrointestinal infection, and multiple forms of cancer are already underway in China, as well as for several forms of cancer in the USA (www.clinicaltrials.gov). In these studies, cells will be removed from the patient and modified with CRISPR, then will be reintroduced into the patient. In addition to the enormous benefit provided by generating hiPSC-based models of disease, in the future it may be possible to introduce patient-derived hiPSCs modified by CRISPR (to introduce corrective or protective mutations) into patients to treat neurodegenerative illnesses. Stem cell transplants have already been used with success in rodent models of neural disease (15, 181–185), which gives hope that human iPSC transplants will soon become a practicable therapy.

## Conclusion

As we have described here, hiPSCs are an enormously powerful tool for neuropsychiatric disease modeling. The ability to generate nearly limitless quantities of patient-derived neurons, combined with CRISPR/Cas9 gene editing, allows researchers to introduce or correct disease-relevant mutations in order to study the pathophysiology of disease risk. 3D culture systems will ultimately yield disease models that more faithfully reflect the complex interconnectivity of the human brain. By studying hiPSC-derived neurons from patients with defined genetic mutations associated with particular illnesses, disease mechanisms can be explored in living tissue on a genetic background specific to the patient. As pathological phenotypes are identified, patient-derived neurons can be used for pharmacological drug screening. For these reasons, hiPSCs provide an invaluable research tool for investigators and a source of hope for patients struggling with neurological and psychiatric diseases.

## Definitions

### Positive, Negative, and Cognitive Symptom Domains of Schizophrenia

Positive symptoms of schizophrenia include functions or behaviors that are not present in healthy individuals, but are present in patients. These include hallucinations, delusions, abnormal movement, and thought disorders. Negative symptoms include functions or behaviors that are not present in patients, which typically are in healthy individuals. Such examples are flat affect, alogia, anhedonia, avolition, and asociality. The cognitive symptom domain includes disruptions in executive function, working memory, attention, and learning.

### Low-Copy Repeat Segments

Segments of highly repetitive DNA can give rise to chromosomal rearrangements.

### Non-Allelic Homologous Recombination

Exchange of genetic material is between similar or identical regions of DNA within different alleles.

### Primary Neural Cultures

Neural cells are derived from the brain *in vivo* (postmortem or biopsy) and cultured *in vitro*.

### First-Generation Antipsychotic

An older class of antipsychotics that primarily act on the DA system, particularly blocking the action of dopamine 2 receptors (e.g., chlorpromazine and haloperidol), and are associated with movement-related extrapyramidal side effects and tardive dyskinesia.

### (Clozapine) Second-Generation Antipsychotic

A newer class of antipsychotics with actions at a variety of neurotransmitter receptors, most notably DA and serotonergic receptors, along with significant antihistaminic and anticholinergic activity (e.g., clozapine, olanzapine, quetiapine, and risperidone). Although second-generation antipsychotics generally cause lower rates of extrapyramidal side effects than do the first generation of antipsychotics, their use is associated with high rates of metabolic adverse effects. In the case of clozapine, the risk of a rare but potentially life-threatening blood condition (agranulocytosis) has limited its use to treatment-refractory cases.

## Author Contributions

EL, SP, and KB conceived the concept and structure of the paper; EL and SP wrote the manuscript; KB critically revised the manuscript; KB and SA supervised the preparation and submission of the manuscript; EL prepared the figures. All the authors reviewed and approved the final manuscript for publication.

## Conflict of Interest Statement

The authors declare that the research was conducted in the absence of any commercial or financial relationships that could be construed as a potential conflict of interest.
